# Muscle Atrophy Marker Expression Differs between Rotary Cell Culture System and Animal Studies

**DOI:** 10.1155/2019/2042808

**Published:** 2019-02-17

**Authors:** Charles P. Harding, Elizabeth Vargis

**Affiliations:** Department of Biological Engineering, Utah State University, Logan, 84322, USA

## Abstract

Muscular atrophy, defined as the loss of muscle tissue, is a serious issue for immobilized patients on Earth and for humans during spaceflight, where microgravity prevents normal muscle loading.* In vitro* modeling is an important step in understanding atrophy mechanisms and testing countermeasures before animal trials. The most ideal environment for modeling must be empirically determined to best mimic known responses* in vivo*. To simulate microgravity conditions, murine C2C12 myoblasts were cultured in a rotary cell culture system (RCCS). Alginate encapsulation was compared against polystyrene microcarrier beads as a substrate for culturing these adherent muscle cells. Changes after culture under simulated microgravity were characterized by assessing mRNA expression of MuRF1, MAFbx, Caspase 3, Akt2, mTOR, Ankrd1, and Foxo3. Protein concentration of myosin heavy chain 4 (Myh4) was used as a differentiation marker. Cell morphology and substrate structure were evaluated with brightfield and fluorescent imaging. Differentiated C2C12 cells encapsulated in alginate had a significant increase in MuRF1 only following simulated microgravity culture and were morphologically dissimilar to normal cultured muscle tissue. On the other hand, C2C12 cells cultured on polystyrene microcarriers had significantly increased expression of MuRF1, Caspase 3, and Foxo3 and easily identifiable multinucleated myotubes. The extent of differentiation was higher in simulated microgravity and protein synthesis more active with increased Myh4, Akt2, and mTOR. The* in vitro* microcarrier model described herein significantly increases expression of several of the same atrophy markers as* in vivo* models. However, unlike animal models, MAFbx and Ankrd1 were not significantly increased and the fold change in MuRF1 and Foxo3 was lower than expected. Using a standard commercially available RCCS, the substrates and culture methods described only partially model changes in mRNAs associated with atrophy* in vivo*.

## 1. Introduction

Muscle loss from disuse negatively affects quality of life in patients on Earth and remains a significant risk factor to astronaut health despite rigorous exercise programs onboard the International Space Station [1, 2]. Approximately 40-50% of total body mass is skeletal muscle and its loss can induce numerous detrimental physiological changes, including reduced power, lower endurance, and atypical reflex responses [3, 4]. Disuse, reduced protein synthesis, and reduced motor neuron activity all contribute to losing muscle tissue and strength in spaceflight [3]. Atrophy severity varies with microgravity exposure time and anatomical region. Muscle mass loss for short duration missions ranges from 10 to 20%, compared to 30-50% for long duration missions [5, 6]. To reduce these risks, flight protocol at the National Aeronautics and Space Administration (NASA) mandates that crew members exercise on missions lasting over 10 days; however, loss of strength and muscle mass has been reported after only 5 days [7, 8]. Preserving astronaut strength and endurance by limiting muscle atrophy is critical for enabling long duration space travel and exploration. To this end, ground-based modeling of microgravity is a critical step in developing atrophy countermeasures.

Three key mRNAs in muscle loss are muscle RING finger-1 (MuRF1, also called Trim63), muscle atrophy F-box (MAFbx, also called Fbxo32 and Atrogin-1), and Caspase 3, all upregulated in numerous rodent models of muscular atrophy including disease, immobilization, hind limb unloading, and spaceflight [9–22]. MuRF1 and MAFbx are key E3 ubiquitin ligases involved in recycling of muscle proteins [14]. However, intact muscle fibers cannot be degraded by the ubiquitin proteasome system and must be broken down before MuRF1 and MAFbx can act. This breakdown is induced by Caspase 3, upregulated during muscular atrophy and responsible for the degradation of actomyosin complexes in the muscle tissue [4, 23]. Following hind limb unloading, knockout mice lacking MuRF1 and MAFbx display significantly less atrophy than wild type mice, highlighting the importance of those ligases in the mechanisms underlying muscle loss [10, 14].

Additional mRNAs important for monitoring muscle health include Akt2, Foxo3, mTOR, and Ankrd1. Akt2 (also called Protein Kinase B *β*) is a serine/threonine-protein kinase and a downstream target of insulin-like growth factor (IGF) induced muscle differentiation [24]. Upregulated significantly in differentiated skeletal muscle, the absence of Akt2 leads to a substantial reduction in myotube diameter [25]. Forkhead Box O3 (Foxo3), another downstream target of the IGF signaling pathway, is also upregulated during muscle atrophy* in vitro* and* in vivo* [26–28]. Foxo3 is responsible for activation of multiple atrophy-related transcription factors, including the ubiquitin ligase MAFbx [28]. A third component of the IGF signaling pathway is mammalian target of rapamycin (mTOR), a regulator of protein synthesis and muscle hypertrophy that is increased by mechanical stimulation and in the presence of nutrients and growth factors [29, 30]. Unlike the previously discussed mRNAs, mTOR expression decreases during muscle atrophy as the ubiquitin proteasome system becomes more active.

Finally, cardiac ankyrin repeat protein (Ankrd1, also called CARP) is upregulated in both unloading and denervation models* in vivo* [27, 31]. The increase in Ankrd1 expression during muscular atrophy has been reported as up to an order of magnitude higher than that of other markers such as MAFbx and MuRF1 [31]. Furthermore, the aforementioned proteasome-related markers may only be temporarily upregulated during the initial stages of muscle atrophy, where Ankrd1 is persistently expressed at high levels [31]. The large, easily detected increase in Ankrd1 makes it an attractive target for evaluating muscular atrophy models.

A classic method for simulating weightlessness is the hind limb unloading rodent model, developed at NASA in the 1970's [32]. In this model, the rodent is affixed in a harness or tail traction device such that the hind limbs are elevated at a 30° angle [32]. The resulting unloading induces muscle atrophy in the hind limbs and cephalic fluid shift similar to real microgravity conditions [32]. However, ground-based animal models differ from human physiology, are more time consuming and more expensive, and are subject to more regulation than cell culture models, providing strong motivation to develop other methods. Newly developed therapeutics can be effectively screened with smaller quantities in cell culture models and safe dose ranges established prior to testing* in vivo*. Highly tissue-specific effects can be elucidated without confounding variables introduced by other systems in the organism. Additionally, cell culture models enable use of primary human cell lines, increasing biological relevance to humans.

*In vitro* modeling of microgravity can be conducted with rotary cell culture systems (RCCS) and three-dimensional random positioning machines or clinostats [33, 34]. Here, we employ the RCCS, developed by Synthecon Inc. in conjunction with NASA, to simulate microgravity [33]. In the RCCS, microgravity is mimicked by the rotational motion of the vessel maintaining cells at their terminal settling velocity, similar to what astronauts experience in orbit around Earth. The RCCS has been used to simulate microgravity in a variety of cell types, such as lymphocytes, osteoblasts, and myoblasts, including the C2C12 mouse myoblast cell line used herein [35–40]. The C2C12 cell line differentiates into contractile skeletal muscle fibers and produces many of the same proteins and mRNAs as human muscle tissue [41]. Use of a mouse cell line for* in vitro* model development and extension also benefits from a large body of literature available on mRNA expression in live mouse microgravity models, providing information for evaluating the model's similarity to* in vivo* studies. Previously published work with muscle cells, including C2C12s, in simulated microgravity focused on changes in differentiation induced by culture in the RCCS [37–39]. To the best of the authors' knowledge, no previously published work has investigated changes in atrophy-specific mRNAs with muscle cell culture in the RCCS.

Standard culture methods for adherent cells in the RCCS employ a substrate to support growth. Two substrates commonly used in three-dimensional cell culture are microcarrier beads and alginate encapsulation. Microcarriers are an attractive substrate due to ease of scalability for producing large quantities of cells for therapeutic applications [42, 43]. As in standard tissue culture flasks, C2C12 cells differentiate on microcarriers* in vitro.* The beads are available in a wide variety of surface chemistries tailored to specific cell types and culture conditions [42].

Alternatively, adherent cells can be encapsulated within several synthetic or naturally occurring hydrogels [44]. Naturally occurring alginate hydrogel has well established uses for mammalian cell encapsulation due to its low toxicity and gentle gelling conditions [44–49]. Additionally, the high porosity of alginate hydrogels is advantageous for maximizing diffusion rates and ensuring adequate exchange of nutrients and waste products with the surrounding culture media [45]. In contrast with microcarrier beads, which can only be seeded with undifferentiated cells, alginate encapsulation can be performed on both undifferentiated and differentiated muscle cells. The percentage alginate used for encapsulation can be varied between 1.5 and 3% (w/v) depending on the cell type and desired mechanical properties [46–50]. To preserve bead integrity in the dynamic RCCS environment, we elected to encapsulate cells at the upper end of this range to maximize mechanical strength of the beads [45].

Here, we propose a ground-based protocol using C2C12 cells and a RCCS to induce expression of the atrophy-related mRNAs MuRF1, Caspase 3, and Foxo3. Additionally, the model induces significant changes in Akt2 and mTOR expression. The model is limited by a lack of significant MAFbx and Ankrd1 expression and low fold changes in MuRF1 and Foxo3. While not currently suitable for these purposes, with further development we expect that the RCCS can be improved to more closely match the expression changes of animal hind limb unloading models.

## 2. Materials and Methods

### 2.1. Cell Culture

C2C12 (ATCC CRL-1772, Manassas, VA, USA) cell stocks were maintained in their undifferentiated state with Dulbecco's Modified Eagles Medium (DMEM) and 10% fetal bovine serum (FBS) from HyClone, GE Healthcare (Logan, UT, USA). Stocks were passaged at 60-70% confluency during scale-up. Cell counting prior to seeding was performed with a Beckman Coulter Vi-Cell (Indianapolis, IN, USA), which uses a trypan blue exclusion assay. The experimental conditions consisted of four replicated vessels per run for each condition described in Table 1. Controls consisted of standard T25 tissue culture flasks for alginate encapsulated conditions and ultra-low attachment (ULA) tissue culture flasks (Corning, NY, USA) for microcarrier conditions.

The use of ultra-low attachment flasks for microcarrier cultures was necessary to prevent cells from growing on the flask surface, forcing adherence on the microcarrier beads and ensuring the available surface area for cell growth was identical to the RCCS vessels. Cells were seeded into each experimental condition of Table 1 at 2.5x10^5^ cells mL^−1^ with a total volume of 10 mL. Culture conditions were maintained at 37°C and 5% CO_2_. Every 3 days, the culture media was changed for fresh media. On day 6, the media was changed to DMEM 2% FBS to promote differentiation of the myocytes into myotubes. All cultures were maintained for a total of 15 days. The 15-day culture time was selected to maximize exposure to the simulated microgravity environment while preventing loss of cell attachment from microcarriers, previously reported for longer duration cultures [51]. For the final condition in Table 1, the cells were moved from ULA T25s to the RCCS for the final 3 days of the culture period.

### 2.2. Microgravity Simulation

A Synthecon RCCS-4H (Houston, TX, USA) with four 10 mL high-aspect-ratio vessels (HARVs) was used to generate simulated microgravity conditions. Sterilization and operation of the Synthecon RCCS-4H were carried out in accordance with the manufacturer's instructions. Vessel rotation was adjusted empirically to maintain the majority of the substrate in suspension, as described in Table 2. The horizontally orientated RCCS, investigated as a normal gravity control, was maintained at the mean RPM of 15. When the substrates are maintained at their terminal settling velocity, the force of gravity *F*_*g*_ is described by Stoke's Law (Equation (1)). Consider

(1) $$\begin{array}{c} F_{g}=\left(\;\rho_{a}-\rho \;\right)g\frac{4}{3}\pi R^{3} \end{array}$$

In (1), *ρ*_*a*_ is the density of the cell aggregate, *ρ* is the density of the culture media, *R* is the radius of the cell aggregate, and *g* is the force of gravity. The resulting *F*_*g*_ is balanced by the drag force from the rotating fluid, resulting in a net force of zero. Since *g* and *R* are constant at any given moment, the ratio of *F*_*g*_ in the RCCS to the normal force of gravity can be simplified to express how many times Earth's gravity (*Xg*) is produced within the system, as described by (2) and detailed in Table 2.

(2) $$\begin{array}{c} Xg=\frac{F_{g\;RCCS}}{F_{g\;Normal}}=\frac{\rho_{a}-\rho }{\rho_{a}} \end{array}$$

### 2.3. Microcarriers

Undifferentiated C2C12 cells were cultured on 5 mg mL^−1^ of HyClone HyQ Sphere Pplus 102-L microcarrier beads. These polystyrene beads have a cationic surface charge and are well suited for culturing cells in agitated vessels with serum-containing media [42]. Rotation of the culture vessels was initiated at 10 RPM and increased up to 20 RPM as the culture age increased and amalgamations of beads formed. Vessel RPM was adjusted empirically to prevent settling and maintain the beads suspended at their terminal settling velocity. Microcarrier beads in static ULA T25 tissue culture flasks provided a normal-gravity control and produced microcarrier cluster sizes similar to those in the RCCS.

### 2.4. Alginate Encapsulation

Undifferentiated C2C12 cells were mixed with 3% (w/v) solution of high viscosity alginate (MP Biomedicals, Santa Ana, CA, USA) at a density of 10x10^6^ cells mL^−1^. Beads were formed by dripping the alginate-cell mixture into 200 mM CaCl_2_ 10 mM HEPES at pH 7 through a 19-gauge syringe needle at a flow rate of 2 mL min^−1^. The beads were cured in CaCl_2_ buffer for 20 minutes at 37°C. Once cured, the average bead diameter was 3252±103 *μ*m with a volume of approximately 18 *μ*L, yielding 1.8x10^5^ cells per bead. To match the seed density of the microcarriers, 14 alginate beads were used per 10 mL vessel. The beads were rinsed once with DPBS and transferred to their respective culture conditions in DMEM 10% FBS.

Differentiated cells were encapsulated following 9 days of growth in standard tissue culture flasks. The myotubes were removed from the flask with a cell scraper and resuspended at a density of 75 cm^2^ culture area per mL of alginate. Subsequent alginate encapsulation methods were performed as described for undifferentiated cells.

Upon harvest, the alginate beads were dissolved with 100 mM sodium citrate, 150 mM NaCl, 30 mM EDTA at pH 7.0, and 37°C. The cells were pelleted via centrifugation and rinsed with DPBS before proceeding with RNA extraction, as described in the following section.

### 2.5. Atrophy Marker Expression

Relative expression of the atrophy markers MuRF1, MAFbx, and Caspase 3 was determined using RT^2^ qPCR primer assays from Qiagen (Hilden, Germany). Akt2, mTOR, Foxo3, and Ankrd1 primers were purchased from Bio Rad (Hercules, CA, USA). RNA extraction was carried out immediately upon culture harvest using the illustra RNAspin Mini Kit according to the manufacturer's instructions (GE Healthcare, Marlborough, MA, USA). Extracted RNA was quantified with a NanoDrop (Thermo Fisher Scientific, Waltham, MA, USA) and converted into cDNA with the RT^2^ First-Strand Kit from Qiagen. All PCR reactions were loaded with 5 ng of cDNA and SYBR Green ROX qPCR Mastermix from Qiagen. Fluorescence was read with an Eppendorf Mastercycler realplex4 (Hamburg, Germany). Cycle settings were 95°C for 10 minutes, followed by 40 cycles of 95°C for 15 seconds and then 60°C for 1 minute. Analysis was performed using the default noise band threshold with drift correction applied in Eppendorf Mastercycler realplex 2.2 software. All results were normalized to glyceraldehyde 3-phosphate dehydrogenase (GAPDH), a suitable housekeeping gene for atrophying muscle tissue* in vitro* and* in vivo *[52–57]. GAPDH expression in our samples was stable, with a CV of 2.5% for all microcarrier samples and 3.7% for all alginate samples. Normalization was calculated with the 2^ΔΔCt^ method [58].

### 2.6. Microscopy

Brightfield microscopy was performed using an AMG EVOS xl from Thermo Fisher Scientific to assess encapsulated cell distribution and alginate bead size and shape. The beads were then fixed in cold methanol for 30 minutes at −20°C, transferred to a solution of 30% sucrose 0.1 M CaCl_2_ overnight at 4°C and then frozen in Tissue-Plus O.C.T. compound (Fisher Scientific, Pittsburg, PA, USA) at −20°C until sectioning [59]. Using a Leica CM1850 cryomicrotome (Wetzlar, Germany), the beads were sliced into 30 *μ*m sections and mounted on Fisher SuperFrost Plus slides, treated with 1 *μ*M Hoechst 33342 (Invitrogen, Carlsbad, CA, USA) to visualize the nuclei and imaged with an Observer Z1 confocal microscope (Zeiss, Oberkochen, Germany).

Cell morphology for microcarriers was also evaluated via fluorescent imaging with the Observer Z1 confocal microscope. Microcarrier-cell clusters were first rinsed with DPBS and then incubated with 1 *μ*M Hoechst 33342 and 50 nM MitoTracker CMX-Ros (Invitrogen, Carlsbad, CA, USA) for 1 hour at 37°C. The clusters were rinsed again with DPBS and z-stack images were acquired with a 3 *μ*m step size. Diameter of multinucleated muscle fibers growing on the microcarriers was determined with ImageJ.

### 2.7. Protein Assay

Extent of terminal differentiation was quantified by ELISA for mouse myosin heavy chain 4 (Myh4) (Biomatik, Wilmington, DE, USA), present in high levels in fused myotubes [60]. Cells were rinsed with PBS and lysed using a urea-based lysis buffer containing 8 M urea, 300 mM NaCl, 5 mL L^−1^ Triton X-100, 50 mM sodium phosphate dibasic, and 50 mM Tris-HCl. A protease inhibitor cocktail consisting of 1 mM PMSF and 0.1 mg mL^−1^ of pepstatin, antipain, and leupeptin was added to the lysis buffer. The cells were lysed on the microcarriers and the lysate was isolated by centrifugation. Lysate protein content was determined using a BCA protein assay kit (Pierce, Rockford, IL, USA) and a SpectraMax i3x (Molecular Devices, San Jose, CA, USA). Lysates were diluted to 1 *μ*g mL^−1^ total protein with PBS and the ELISA kit ran according to the manufacturer's instructions. Quantitation was performed using the Spectramax i3x.

### 2.8. Statistics

Graphical data are presented as the means ± standard deviation. Significance was determined by comparing data sets with Student's t-test. Differences were considered significant at p<0.05.

## 3. Results

### 3.1. Microcarrier Substrate Structure and Cell Morphology

**Figure 1:**
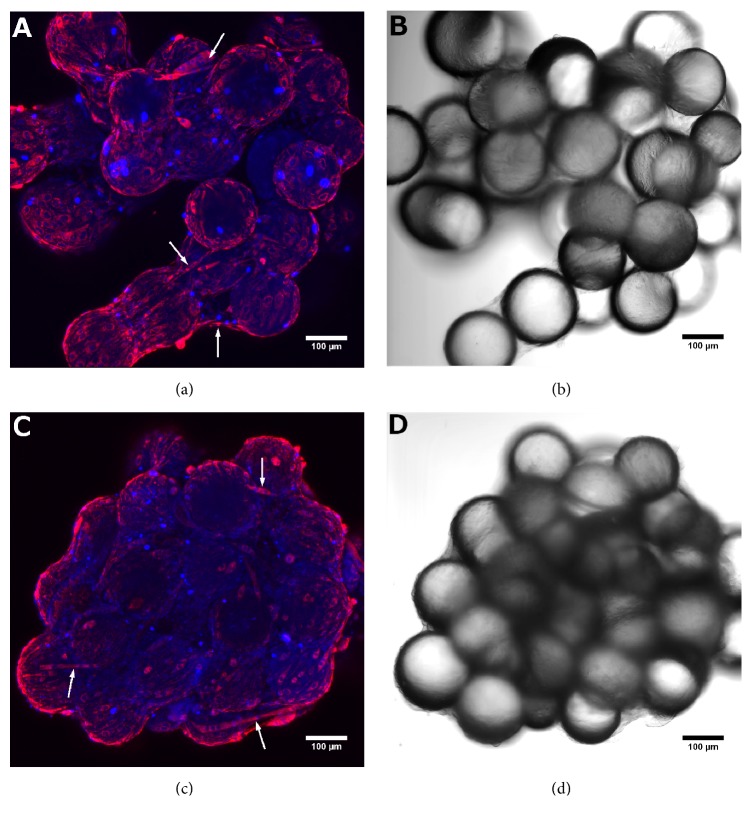
Microcarrier bead clusters after 15 days of culture, imaged at 10x magnification. Nuclei are stained blue with Hoechst 33342. Mitochondria are stained red with MitoTracker CMX-Ros. Normal gravity (a, b) and simulated microgravity (c, d) bead clusters both have differentiated, multinucleated myotubes spanning across bead gaps (white arrows). Scale bar = 100 *μ*m.

When cultured on HyQSphere Pplus 102-L microcarriers, C2C12 cells form monolayers over the bead surface (Figure 1). Differentiated, multinucleated myotubes were observed spanning across beads (Figures 1(a) and 1(c)). No significant difference was observed in differentiation or number of multinucleated myotubes between simulated microgravity and normal gravity controls. The irregular three-dimensional structure of the bead amalgamations was best visualized with brightfield microscopy (Figures 1(b) and 1(d)). Microcarrier bead cluster size increased with culture age in both normal gravity controls and simulated microgravity conditions. Due to the lack of agitation, maximum cluster size was larger in normal gravity controls than in the RCCS. Additionally, bead clusters formed in normal gravity had widely varying, irregular shapes compared to consistently rounded bead clusters formed in the RCCS (Figures 1(a) and 1(b) versus Figures 1(c) and 1(d)). Despite variations in cluster size and shape, no significant differences in cell morphology were observed. Muscle fiber diameter was 12.7±6.0 *μ*m and 9.5±1.7 *μ*m for normal gravity controls and simulated microgravity conditions, respectively. Fiber diameters were more consistent in the RCCS, but the difference between conditions was not significant (p>0.05, n=10).

### 3.2. Alginate Substrate Structure and Cell Morphology

**Figure 2:**
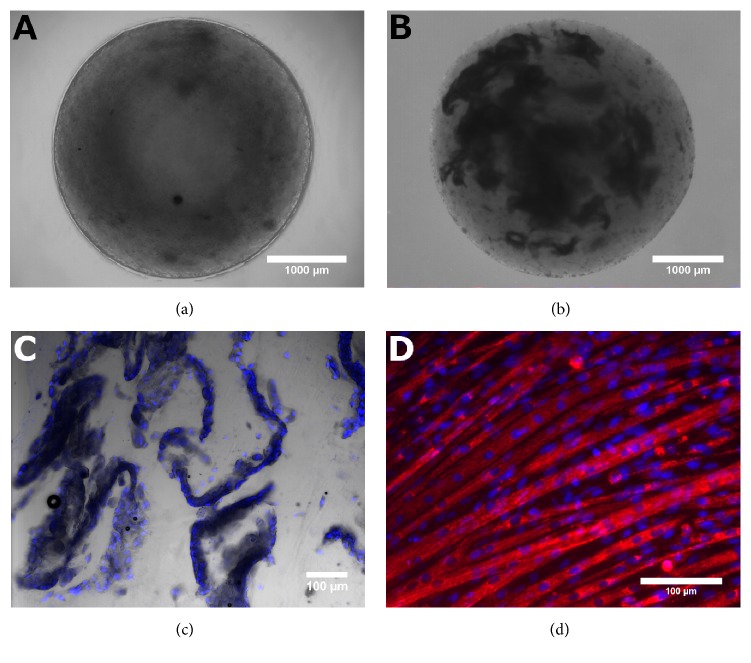
Alginate encapsulated undifferentiated (a) and differentiated cells (b) imaged at 2x magnification, scale bar = 1000 *μ*m. Interior morphology of encapsulated differentiated cells in a 30 *μ*m section of an alginate bead (c) compared to a standard T25 (d), scale bar = 100 *μ*m. Nuclei are stained blue with Hoechst 33342 ((c) & (d)). Mitochondria are stained red with MitoTracker CMX-Ros ((d) only).

Undifferentiated cells encapsulated in alginate were evenly distributed throughout the bead (Figure 2(a)), where encapsulation of differentiated cells resulted in irregular sheets with uneven distribution (Figure 2(b)). A section of an alginate bead containing differentiated cells reveals the encapsulation process forces the harvested muscle fibers into atypical configurations (Figure 2(c)), compared to the regular, parallel orientation common in standard tissue culture flasks (Figure 2(d)). For cells encapsulated in their undifferentiated state, minimal differentiation was observed after 15 days of culture. No differences in cell morphology were observed between the RCCS and normal gravity controls. Alginate bead degradation was minimal, as more than 90% of the beads were intact when they were harvested at the end of the culture period.

### 3.3. Atrophy Marker Expression

**Figure 3:**
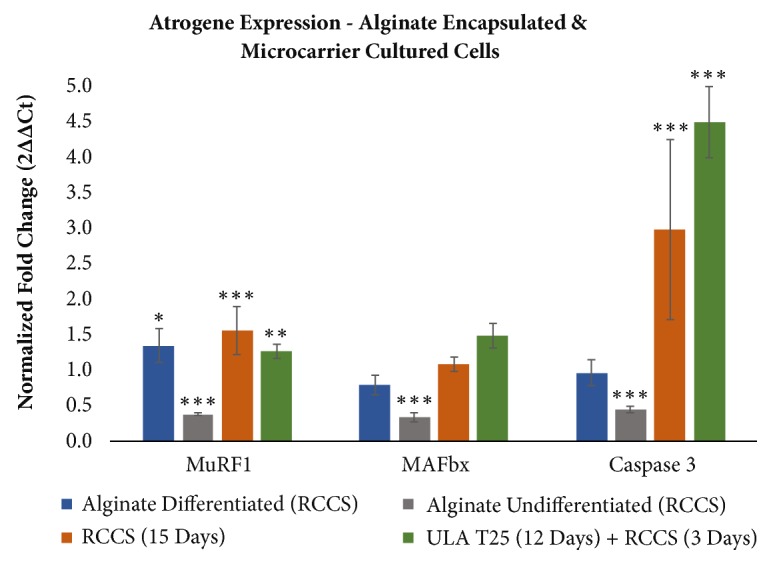
Average ± s.d. fold change in expression of mRNAs for alginate and microcarrier cultures, relative to their respective normal gravity controls (alginate N=4, microcarrier N=20) and normalized by GAPDH. *∗* p<0.05, *∗∗* p<0.01, and *∗∗∗* p<0.001. No marker = not significant. N=4 (alginate differentiated), N=4 (alginate undifferentiated), N=16 (RCCS), and N=8 (ULA T25 + RCCS). All replicates are biological replicates and significance was determined by* t*-test.

Alginate-encapsulated differentiated cells in the RCCS expressed significantly more MuRF1 than those in T25s, but the difference in MAFbx and Caspase 3 expression was not significant (Figure 3). Encapsulated undifferentiated cells in the RCCS expressed significantly less MuRF1, MAFbx, and Caspase 3 than those in T25s, contrary to the hypothesized response (Figure 3).

When cultured on microcarriers, expression of MuRF1 and Caspase 3 was significantly elevated after 12 days in ULA T25s, followed by 3 days in the RCCS, relative to ULA T25 control flasks (Figure 3). Cultures that remained in the RCCS for 15 days also expressed significantly more MuRF1 and Caspase 3 than the ULA T25 control. The change in MAFbx was not significant for either simulated microgravity culture method but was larger for the ULA T25 (12 days) + RCCS (3 days) condition (p=0.1). Significance for all comparisons is detailed in Table 3.

**Figure 4:**
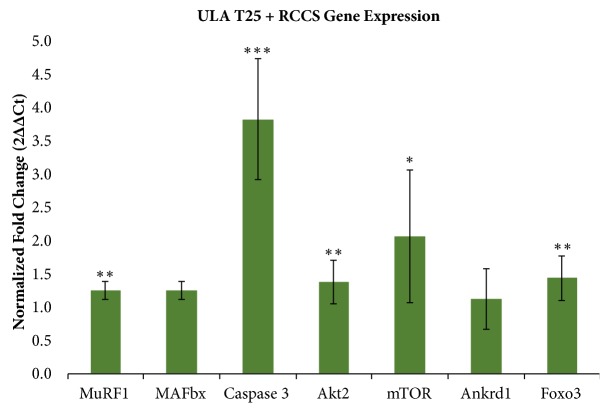
Average ± s.d. fold change in expression of mRNAs for ULA T25 + RCCS simulated microgravity cultures (N=8) relative to ULA T25 normal gravity control (N=20) and normalized by GAPDH. *∗* p<0.05, *∗∗* p<0.01, and *∗∗∗* p<0.001. All replicates are biological replicates and significance was determined by* t*-test. Change in MAFbx and Ankrd1 not significant.

Cells cultured in the RCCS for 15 days and those cultured in the RCCS for only 3 days following 12 days in ULA T25s had similarly significant increases in the key atrophy markers MuRF1 and Caspase 3. Due to the shorter duration of RCCS culture, using the latter method allows for higher throughput testing in a given timeframe, as subsequent cultures need only be staggered by 3 days instead of 15. Therefore, the ULA T25 + RCCS method was selected for further development of the model and evaluation of Akt2, mTOR, Ankrd1, and Foxo3. Akt2, mTOR, and Foxo3 increased significantly in simulated microgravity, but the change in Ankrd1 was not significant (Figure 4).

### 3.4. Protein Concentration

Myosin heavy chain 4 (Myh4) was used to assess the extent of terminal differentiation. After 15 days of culture on microcarriers in ULA normal gravity control flasks, Myh4 concentration was 1.22±0.26 ng *μ*g^−1^ total protein. Cells cultured in the RCCS for the final 3 days following 12 days in the ULA flasks had an Myh4 concentration of 2.13±0.48 ng *μ*g^−1^ total protein. The difference between the normal gravity and simulated microgravity conditions was significant (p<0.05, N=4).

## 4. Discussion

We hypothesized that alginate encapsulated cells would exhibit more significant increases in atrophy marker expression than cells cultured on microcarriers, since an encapsulated three-dimensional mass of differentiated muscle tissue may be more similar to animal models than monolayers on the exterior of microcarriers. However, the alginate-encapsulated differentiated cells produced an increase in MuRF1 only. Further, encapsulated undifferentiated cells had significantly lower expression of MuRF1, MAFbx, and Caspase 3 in simulated microgravity, which was the opposite of expected results (Figure 3). Low expression of atrophy markers for encapsulated undifferentiated cells may be due to insufficient myotube formation. The solid alginate structure limits cell-cell contact and morphology remained consistent over the 15-day culture period (Figure 2(a)). On the other hand, C2C12 cells cultured on polystyrene microcarrier beads for 12 days in ULA T25 flasks followed by 3 days in the RCCS significantly (p<0.05) express more MuRF1, Caspase 3, Akt2, mTOR, and Foxo3 than the normal gravity control of cells cultured in static ULA T25s for all 15 days, highlighting the early upregulation of these markers in simulated microgravity (Figure 4).

The timing of maximum MAFbx and MuRF1 expression* in vivo* remains under investigation. Elevated expression has been reported at 3 days, 7 days, and 6 weeks, though differences in animal line and immobilization methods may be responsible [19, 21, 22]. Unlike MAFbx and MuRF1,* in vivo* atrophy models produce consistent significant increases in Caspase 3 over 5, 10, and 14 days [17, 18]. Our results* in vitro* indicate that MuRF1 expression is higher in cells cultured for 15 days in the RCCS, compared to 3 days in the RCCS after 12 days in ULA flasks, while Caspase 3 expression does not change significantly (Figure 3 and Table 3). We did not observe a significant change in MAFbx at either 3 or 15 days in simulated microgravity, suggesting that the timing of upregulation differs from its partner E3 ligase MuRF1. In addition to the uncertain timing of peak upregulation, this discrepancy in MuRF1 and MAFbx upregulation may be due to the different roles of the two ligases. While MuRF1 and MAFbx both play an important part in the ubiquitin proteasome system, MuRF1 is more closely associated with degradation of myofibers, where MAFbx attenuates new protein synthesis and is not correlated with atrophy in every case [61, 62].

Increased expression of Akt2 and higher Myh4 concentrations in simulated microgravity indicate a higher percentage of differentiated cells compared to the normal gravity controls. mTOR, expected to be lower in simulated microgravity, instead increased significantly after 3 days in the RCCS (Figure 4). Additionally, while Foxo3 will activate MAFbx, Foxo3 was significantly upregulated while MAFbx—responsible for attenuating protein synthesis—was not (Figure 4) [28, 61]. Taken together, these results indicate that while the cells in simulated microgravity were undergoing atrophy—indicated by MuRF1, Caspase 3, and Foxo3—mTOR mediated protein synthesis and formation of new myotubes had not yet ceased.

Compared to* in vivo* methods, including casting, tenotomy, and hind limb unloading, our* in vitro* model resulted in a similar fold change in Caspase 3 and lower but still statistically significant increases in MuRF1 and Foxo3 (Table 4). While Caspase 3 is most strongly upregulated, apoptosis from shear forces is not likely due to the low shear environment of the RCCS [33]. The fold increase in MuRF1 after 12 days in normal gravity and 3 days in the RCCS was closer to that of 3 days after tenotomy rather than 3 days after immobilization via casting (Table 4). The* in vitro* model did not significantly increase MAFbx, but again the fold change was closer to that following tenotomy. In contrast to the large increase in Ankrd1 seen* in vivo*, our* in vitro* model did not produce any significant changes (Table 4). While Ankrd1 is expressed in C2C12 cells, it was not significantly upregulated in this model [63, 64].

Turning the RCCS horizontal was investigated as a normal gravity control to account for the effects related to vessel geometry and fluid motion not present in static ULA tissue culture flasks. Despite these benefits, the horizontal RCCS was not an ideal control due to significant differences in microcarrier bead cluster morphology and higher MuRF1 expression levels compared with the simulated microgravity conditions (RCCS, ULA T25 + RCCS) (not shown). Additionally, microcarrier beads in the horizontal RCCS adhered to the silicone gas transfer membrane, resulting in large sheets of cells not representative of the bead clusters in Figure 1. Shear forces from fluid motion in the RCCS are minimal and selecting a control with similar cluster morphology and consistently significant differences in mRNA expression is preferred [33].

Microcarrier beads and alginate encapsulation each have advantages and disadvantages in maximizing similarity with* in vivo* hind limb unloading methods. The advantages of microcarriers include ease of handling, live cell fluorescent imaging capability, and, most importantly, that they support atrophy marker changes in C2C12 cells. Culture procedures with microcarrier beads are simpler and more rapid than alginate encapsulation. The small size of the beads allows them to be transferred between containers via pipette. Harvest of RNA can be accomplished by adding the cell-covered microcarriers directly to the lysis buffer, reducing process time and opportunity for RNase activity. Most critically, simulated microgravity culture of C2C12 cells on microcarriers resulted in significant expression changes in multiple atrophy markers compared to the normal gravity control (Figure 4). However, MAFbx and Ankrd1 were not significantly upregulated and the fold increase in MuRF1 and Foxo3 was low, indicating that this model is incomplete when compared to atrophy modeling* in vivo* (Table 4).

Microcarriers carry some additional disadvantages regarding the culture method and morphology. The monolayer of tissue that forms over the bead surfaces does not replicate three-dimensional mature tissue* in vivo*. Compared to astronauts that begin with differentiated muscle tissue, microcarriers are first seeded with undifferentiated myoblasts which form multinucleated myotubes during culture. Despite this difference, culture with undifferentiated cells remains clinically relevant as fusion and differentiation of muscle cells are important parts of myogenesis in adults [65]. Another disadvantage of microcarriers is the large variations in cluster diameter relative to alginate beads (Table 2). As settling rate within the RCCS varies with substrate diameter, a portion of the microcarrier clusters will not be maintained at the optimal settling rate to simulate microgravity. Additionally, bead cluster size increases over time, requiring daily monitoring and RPM adjustment.

While alginate did not support the desired changes in atrophy marker expression, it offers some beneficial properties compared to microcarriers. Alginate encapsulation of differentiated cells in a three-dimensional conformation improves morphological biosimilarity to mature tissue. However, the current implementation resulted in muscle fiber conformations not typical* in vivo* (Figure 2(c)). Encapsulation also protects cells from exposure to mechanical stress within the RCCS. Fluid flow in the RCCS is designed to provide a low shear environment [33]. Still, cells experience mechanical stress from collisions of suspended substrates due to the rotation of the system. While cells encapsulated in alginate are protected from direct contact, the exposed nature of cells cultured on microcarriers means that collisions between beads will result in a direct impact to the cells. Furthermore, alginate beads have a more consistent size than microcarrier bead clusters and did not display the same variations in settling rate or require changes in the RCCS's RPM (Table 2).

The downsides of alginate, in addition to the lack of significant changes in atrophy marker expression, stem from their density, size, and opacity. While the solid structure of alginate protects cells from mechanical stress, it also limits the spreading of encapsulated cells, preventing formation of intercellular junctions necessary for myoblast fusion (Figure 2(a)). In contrast to the small and easy-to-handle microcarriers, alginate beads, with a diameter of 3252±103 *μ*m, were too large to be aspirated into a pipette. Smaller alginate beads can be formed by using a higher gauge needle, but narrower needles were clogged by the large sheets of differentiated muscle tissue seen in Figure 2(b). The large bead size also complicated imaging due to the opacity of alginate, which scattered too much light to allow fluorescent imaging of the interior without fixation and sectioning. While these issues are addressable with process modifications, the most significant issue is that nutrient and waste diffusion rates are dependent on depth, such that beads with tissues closer to the surface may behave differently than those with tissues encapsulated in the center. As the position of tissues within the bead is random, we attribute some of the variability in encapsulated differentiated samples to uneven distribution of tissues within the alginate beads.

Overall, culturing with microcarrier beads provides a better platform than alginate encapsulation for modeling muscular atrophy in C2C12 cells, though this model does not completely mimic the mRNA changes seen* in vivo*. Cultures on microcarrier beads resulted in irregular clusters covered with a monolayer of cells and sporadic myotubes (Figure 1) and appropriate atrophy marker expression to mimic* in vivo* studies only with regard to MuRF1, Caspase 3, and Foxo3, albeit with lower—but still significant—fold changes for MuRF1 and Foxo3 (Table 4). On the other hand, cultures with encapsulated cells resulted in static, regularly dispersed undifferentiated cells (Figure 2(a)) or unevenly dispersed sheets of differentiated tissue (Figure 2(b)) without consistent increases in expression of the selected atrophy markers. Therefore, we conclude that an* in vitro* model of microgravity-induced muscle atrophy using polystyrene microcarrier beads is superior to alginate encapsulation for cultured C2C12 cells, but not sufficient to mimic all of the select atrophy markers as in animal studies. Of the culture methods evaluated, the most significant and consistent increase in the selected atrophy markers, relative to GAPDH (Figure 4), was achieved with microcarriers cultured in static ULA flasks for 12 days and then moved to the RCCS for the final 3 days.

Our verification that the RCCS can produce increases in atrophy-specific mRNAs in cultured muscle cells is an important step towards completing a comprehensive ground-based* in vitro* model for spaceflight atrophy. Due to the lower number of atrophy markers upregulated and the lower fold changes in expression, relative to animal models, we believe the standard commercially available RCCS is not sufficient for use* in vitro* atrophy modeling and it does not match results* in vivo*. Nevertheless, our results* in vitro* are highly significant for MuRF1 (p<0.01), Foxo3 (p<0.01), and Caspase 3 (p<0.001), indicating that, with further development, simulated microgravity systems may present a promising platform for investigation of atrophy pathways and first-step design and selection of novel therapeutics necessary to ensure astronaut health and fitness during long-term spaceflight.

## Figures and Tables

**Table 1:** Experimental conditions. All cultures had a total duration of 15 days.

Substrate	Cell State at Seeding	Culture Vessel
Alginate	Undifferentiated	Standard T25 (Control)
Alginate	Undifferentiated	RCCS
Alginate	Differentiated	Standard T25 (Control)
Alginate	Differentiated	RCCS

Microcarrier	Undifferentiated	ULA T25 (Control)
Microcarrier	Undifferentiated	RCCS
Microcarrier	Undifferentiated	Horizontally orientated RCCS (Control)
Microcarrier	Undifferentiated	ULA T25 Days 1-12, RCCS Days 13-15

**Table 2:** The RCCS rotation rate necessary to maintain the substrates in suspension varies with diameter. Times Earth gravity (*Xg*) varies with substrate density. ^a^Microcarrier initial diameter at seeding. ^b^Microcarrier cluster diameter at harvest. Data displayed as mean ± CV, n=10.

Substrate	Density (g cm^−3^)	*Xg*	Diameter (*μ*m)	RPM
Microcarriers	1.02	0.02	160±18.7%^a^ to 698±10.5%^b^	10-20
Alginate	1.07	0.07	3252±3.2%	35

**(a):** 

**MuRF1**	ULA T25	RCCS	Horiz. RCCS	ULA T25 + RCCS
ULA T25	—	*∗∗∗*	*∗∗∗*	*∗∗*
RCCS	*∗∗∗*	—	ns	*∗∗*
Horiz. RCCS	*∗∗∗*	ns	—	*∗∗∗*
ULA T25 + RCCS	*∗∗*	*∗∗*	*∗∗∗*	—

**(b):** 

**MAFbx**	ULA T25	RCCS	Horiz. RCCS	ULA T25 + RCCS
ULA T25	—	ns	ns	ns
RCCS	ns	—	*∗∗*	*∗∗∗*
Horiz. RCCS	ns	*∗∗*	—	*∗∗∗*
ULA T25 + RCCS	ns	*∗∗∗*	*∗∗∗*	—

**(c):** 

**Caspase 3**	ULA T25	RCCS	Horiz. RCCS	ULA T25 + RCCS
ULA T25	—	*∗∗∗*	*∗∗∗*	*∗∗∗*
RCCS	*∗∗∗*	—	ns	ns
Horiz. RCCS	*∗∗∗*	ns	—	*∗*
ULA T25 + RCCS	*∗∗∗*	ns	*∗*	—

**Table 4:** Fold change in gene expression of atrophy-indicating mRNAs from this *in vitro* study (ULA T25 12 days + RCCS 3 days) compared to referenced work *in vivo*, as measured by qPCR. ns = not significant, *∗∗* p<0.01, *∗∗∗* p<0.001.

	*In Vitro*	*In Vivo*	*In Vivo *method
MuRF1	1.3*∗∗*	1.9, 1.6 [22]	Casting 3 days, tenotomy 3 days
MAFbx	1.3^ns^	2.7, 1.5 [22]	Casting 3 days, tenotomy 3 days
Caspase 3	3.8*∗∗∗*	1.0, 3.3 [22]	Casting 3 days, tenotomy 3 days
Ankrd1	1.1^ns^	3.9 [27]	Hind limb unloading 6 days
Foxo3	1.4*∗∗*	1.6 [27]	Hind limb unloading 6 days

## Data Availability

The raw mRNA expression and image data used to support the findings of this study are available from the corresponding author upon request.

## Abbreviations

Akt2: RAC-beta serine/threonine-protein kinase
Ankrd1: Cardiac ankyrin repeat protein
C2C12: Mouse myoblast cells
CV: Coefficient of variation
DMEM: Dulbecco's modified eagles medium
DPBS: Dulbecco's phosphate buffered saline
EDTA: Ethylenediaminetetraacetic acid
FBS: Fetal bovine serum
Foxo3: Forkhead box O3
HEPES: 4-(2-Hydroxyethyl)-1-piperazineethanesulfonic acid
MAFbx: Muscle atrophy F-box
mTOR: Mammalian target of rapamycin
MuRF1: Muscle RING finger-1
Myh4: Myosin heavy chain 4
NASA: National Aeronautics and Space Administration
PMSF: Phenylmethylsulfonyl fluoride
RCCS: Rotary cell culture system
